# (*S*)-3-Hydroxybutyryl-CoA Dehydrogenase From the Autotrophic 3-Hydroxypropionate/4-Hydroxybutyrate Cycle in *Nitrosopumilus maritimus*

**DOI:** 10.3389/fmicb.2021.712030

**Published:** 2021-07-05

**Authors:** Li Liu, Daniel M. Schubert, Martin Könneke, Ivan A. Berg

**Affiliations:** ^1^Institute for Molecular Microbiology and Biotechnology, University of Münster, Münster, Germany; ^2^Department of Microbiology, Faculty of Biology, University of Freiburg, Freiburg, Germany; ^3^Marine Archaea Group, MARUM Center for Marine Environmental Sciences, University of Bremen, Bremen, Germany; ^4^Benthic Microbiology, Institute for Chemistry and Biology of the Marine Environments, University of Oldenburg, Oldenburg, Germany

**Keywords:** autotrophy, 3-hydroxypropionate/4-hydroxybutyrate cycle, *Nitrosopumilus maritimus*, ammonia-oxidizing archaea, *Metallosphaera sedula*, 3-hydroxybutyryl-CoA dehydrogenase

## Abstract

Ammonia-oxidizing archaea of the phylum Thaumarchaeota are among the most abundant organisms that exert primary control of oceanic and soil nitrification and are responsible for a large part of dark ocean primary production. They assimilate inorganic carbon via an energetically efficient version of the 3-hydroxypropionate/4-hydroxybutyrate cycle. In this cycle, acetyl-CoA is carboxylated to succinyl-CoA, which is then converted to two acetyl-CoA molecules with 4-hydroxybutyrate as the key intermediate. This conversion includes the (*S*)-3-hydroxybutyryl-CoA dehydrogenase reaction. Here, we heterologously produced the protein Nmar_1028 catalyzing this reaction in thaumarchaeon *Nitrosopumilus maritimus*, characterized it biochemically and performed its phylogenetic analysis. This NAD-dependent dehydrogenase is highly active with its substrate, (*S*)-3-hydroxybutyryl-CoA, and its low *K*_*m*_ value suggests that the protein is adapted to the functioning in the 3-hydroxypropionate/4-hydroxybutyrate cycle. Nmar_1028 is homologous to the dehydrogenase domain of crotonyl-CoA hydratase/(*S*)-3-hydroxybutyryl-CoA dehydrogenase that is present in many Archaea. Apparently, the loss of the dehydratase domain of the fusion protein in the course of evolution was accompanied by lateral gene transfer of 3-hydroxypropionyl-CoA dehydratase/crotonyl-CoA hydratase from Bacteria. Although (*S*)-3-hydroxybutyryl-CoA dehydrogenase studied here is neither unique nor characteristic for the HP/HB cycle, Nmar_1028 appears to be the only (*S*)-3-hydroxybutyryl-CoA dehydrogenase in *N. maritimus* and is thus essential for the functioning of the 3-hydroxypropionate/4-hydroxybutyrate cycle and for the biology of this important marine archaeon.

## Introduction

**FIGURE 1:**
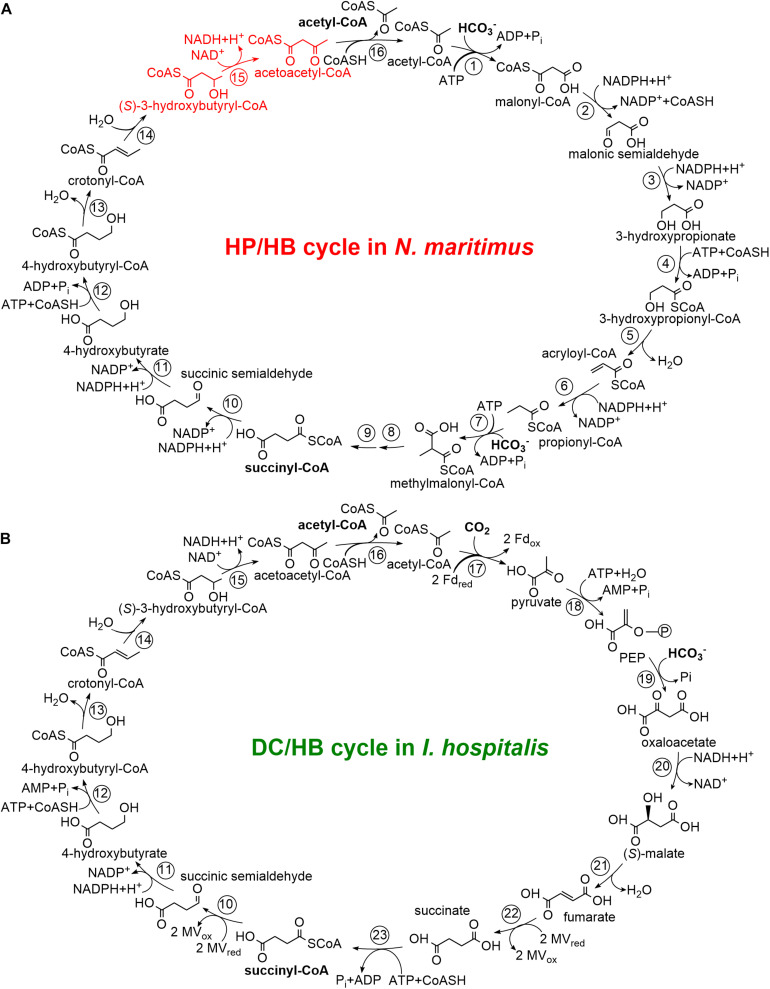
The *N. maritimus* 3-hydroxypropionate/4-hydroxybutyrate cycle **(A)** and the *I. hospitalis* dicarboxylate/4-hydroxybutyrate cycle **(B)** adapted from Berg et al. (2010b) and Könneke et al. (2014). Enzymes: 1, acetyl-CoA carboxylase; 2, malonyl-CoA reductase; 3, malonic semialdehyde reductase; 4, 3-hydroxypropionyl-CoA synthetase; 5, 3-hydroxypropionyl-CoA dehydratase; 6, acryloyl-CoA reductase; 7, propionyl-CoA carboxylase; 8, methylmalonyl-CoA epimerase; 9, methylmalonyl-CoA isomerase; 10, succinyl-CoA reductase; 11, succinic semialdehyde reductase; 12, 4-hydroxybutyryl-CoA synthetase; 13, 4-hydroxybutyryl-CoA dehydratase; 14, crotonyl-CoA hydratase; 15, (*S*)-3-hydroxybutyryl-CoA dehydrogenase; 16, acetoacetyl-CoA β-ketothiolase; 17, pyruvate synthase; 18, PEP synthase; 19, PEP carboxylase; 20, malate dehydrogenase; 21, fumarate hydratase; 22, fumarate reductase; 23, succinyl-CoA synthetase. CoASH, coenzyme A; PEP, phosphoenolpyruvate; Fd, ferredoxin; MV, methyl viologen.

Biological inorganic carbon fixation, the oldest and quantitatively most important biosynthetic process in Nature, proceeds via at least seven natural pathways (Berg, 2011; Fuchs, 2011; Sánchez-Andrea et al., 2020). Two of these pathways, the 3-hydroxypropionate/4-hydroxybutyrate (HP/HB) and the dicarboxylate/4-hydroxybutyrate (DC/HB) cycles were found only in Archaea (Berg et al., 2010a). In both of these cycles, acetyl-CoA is carboxylated to succinyl-CoA, which is then reduced to two acetyl-CoA molecules, regenerating the inorganic carbon acceptor and forming the final product of the cycle(s) (Figure 1). However, the carboxylation reactions are different. The carboxylases in the DC/HB cycle are ferredoxin-dependent pyruvate synthase and phosphoenolpyruvate carboxylase (Huber et al., 2008; Ramos-Vera et al., 2009), while a promiscuous acetyl-CoA/propionyl-CoA carboxylase catalyzes both acetyl-CoA and propionyl-CoA carboxylations in the HP/HB cycle (Chuakrut et al., 2003; Hügler et al., 2003). In the latter case, a number of specific enzymes are responsible for the conversion of malonyl-CoA, the product of the acetyl-CoA carboxylase reaction, to propionyl-CoA, which is then carboxylated and isomerized to succinyl-CoA (Figure 1). Further metabolism of succinyl-CoA is identical in both cycles. Succinyl-CoA reduction leads to the formation of 4-hydroxybutyrate, which is converted to 4-hydroxybutyryl-CoA and dehydrated to crotonyl-CoA. Crotonyl-CoA conversion to two acetyl-CoA molecules proceeds through the reactions known from β-oxidation: hydration of crotonyl-CoA to (*S*)-3-hydroxybutyryl-CoA, its oxidation to acetoacetyl-CoA and cleavage to two acetyl-CoA molecules (Figure 1).

The DC/HB cycle functions in anaerobic Crenarchaeota of the orders Desulfurococcales and Thermoproteales (Huber et al., 2008; Ramos-Vera et al., 2009; Berg et al., 2010b), while the HP/HB cycle is present in aerobic Sulfolobales (Crenarchaeota) and ammonia-oxidizing Thaumarchaeota (AOA) (Ishii et al., 1997; Berg et al., 2007, 2010a; Könneke et al., 2014). For both cycles, variants exist. Although it appears that the cycles in Thermoproteales and Desulfurococcales evolved from a common ancestor, the representatives of these two orders use a number of phylogenetically unrelated enzymes (e.g., succinyl-CoA reductase, succinic semialdehyde reductase) (Huber et al., 2008; Ramos-Vera et al., 2009; Flechsler et al., 2021). For the HP/HB cycle, two fundamentally different variants function in Cren*-* and in Thaumarchaeota. The thaumarchaeal variant uses ADP/P_*i*_-producing 3-hydroxypropionyl-CoA and 4-hydroxybutyryl-CoA synthetases, while non-homologous synthetases produce AMP and PP_*i*_ in the crenarchaeal HP/HB cycle in the corresponding reactions (Berg et al., 2007; Hawkins et al., 2013, 2014; Könneke et al., 2014). The usage of ADP-producing synthetases makes the thaumarchaeal HP/HB cycle the energetically most efficient aerobic autotrophic pathway (Könneke et al., 2014). The characteristic enzymes of these two variants of the cycle are mostly phylogenetically unrelated, implying the involvement of convergent evolution in their emergence (Berg et al., 2007; Könneke et al., 2014; Otte et al., 2015).

Both DC/HB and HP/HB cycles (and all their variants) have in common that crotonyl-CoA is converted to acetoacetyl-CoA (Figure 1). The corresponding enzymes are widespread in prokaryotes, being an interesting example of the involvement of convergent evolution and vertical gene transfer in the emergence of different autotrophic pathways, and a possible role of lateral gene transfer in this process. In the DC/HB cycle, this conversion is catalyzed by a bifunctional crotonyl-CoA hydratase/(*S*)-3-hydroxybutyryl-CoA dehydrogenase consisting of two domains, a C-terminal enoyl-CoA hydratase domain and an N-terminal dehydrogenase domain (Ramos-Vera et al., 2011; Flechsler et al., 2021). This protein is widespread among Archaea and was probably present in the last common ancestor of the TACK-superphylum consisting of **T** haum-, **A** ig-, **C** ren-, and **K**orarchaeota (Williams et al., 2017). Sulfolobales also possess a homologous crotonyl-CoA hydratase/(*S*)-3-hydroxybutyryl-CoA dehydrogenase. Although this protein is present in autotrophically grown *Metallosphaera sedula* (Sulfolobales) cells (Ramos-Vera et al., 2011; Liu et al., 2021), it is responsible only for a minute flux in the crotonyl-CoA conversion to acetoacetyl-CoA in this archaeon (Liu et al., 2020, 2021). In contrast to the organisms using the DC/HB cycle, *M. sedula* and other Sulfolobales possess stand-alone crotonyl-CoA hydratase and (*S*)-3-hydroxybutyryl-CoA dehydrogenase enzymes in the HP/HB cycle (Liu et al., 2020). Their crotonyl-CoA hydratase is a promiscuous protein that has also a high 3-hydroxypropionyl-CoA dehydratase activity and thus catalyzes two (de)hydratase reactions in the cycle (Liu et al., 2021). Thaumarchaeota also use a similar promiscuous enzyme to catalyze these two reactions (Liu et al., 2021). Interestingly, crenarchaeal and thaumarchaeal 3-hydroxypropionyl-CoA dehydratases/crotonyl-CoA hydratases are closely related to bacterial enoyl-CoA hydratases but were retrieved independently by the ancestors of Sulfolobales and AOA from different bacteria (Liu et al., 2021). Here, we characterize (*S*)-3-hydroxybutyryl-CoA dehydrogenase from the ammonia-oxidizing thaumarchaeon *Nitrosopumilus maritimus*, compare it with the enzyme from *M. sedula* and with the crotonyl-CoA hydratase/(*S*)-3-hydroxybutyryl-CoA dehydrogenases, and discuss the enzymological differences in these conversions in course of the DC/HB and HP/HB cycles.

## Materials and Methods

### Materials

Chemicals and biochemicals were obtained from AppliChem (Darmstadt, Germany), Cell Signaling Technology (Frankfurt, Germany), Fluka (Neu-Ulm, Germany), IBA (Göttingen, Germany), Merck (Darmstadt, Germany), Roth (Karlsruhe, Germany), Sigma-Aldrich (Deisenhofen, Germany), or VWR (Darmstadt, Germany). Materials for cloning and expression were purchased from Bioline (London, United Kingdom), MBI Fermentas (St. Leon-Rot, Germany), New England Biolabs (Frankfurt, Germany), or Novagen (Schwalbach, Germany). Materials and equipment for protein purification were obtained from Cytiva (Freiburg, Germany), Macherey-Nagel (Düren, Germany), Millipore (Eschborn, Germany), Pall Corporation (Dreieich, Germany), or Thermo Scientific (Rockford, United States). Primers were synthesized by Sigma-Aldrich (Steinheim, Germany).

### Microbial Strains and Growth Conditions

*N. maritimus* strain SCM1 was cultivated at 28°C in 15-l HEPES-buffered medium (pH 7.5) as previously described (Martens-Habbena et al., 2009). Cells were stored at −20°C and used for DNA isolation. *Escherichia coli* strain TOP10 and *E. coli* strain Rosetta 2 (DE3) (Merck, Darmstadt, Germany) were cultivated at 37°C in lysogeny broth (LB) medium.

### CoA-Esters Synthesis

(*S*)- and (*R*)-3-hydroxybutyryl-CoA were synthesized from the corresponding free acids by the mixed anhydride method (Stadtman, 1957) and then were purified using high-performance liquid chromatography (Zarzycki et al., 2009). Acetoacetyl-CoA and acetyl-CoA were chemically synthesized from diketene and acetic anhydride, respectively, as reported before (Simon and Shemin, 1953). 3-Hydroxypropionyl-CoA was synthesized enzymatically with recombinant propionate CoA-transferase from *Clostridium propionicum* (Selmer et al., 2002).

### Gene Cloning

Chromosomal DNA of *N. maritimus* was extracted using an illustra bacteria genomicPrep Mini Spin Kit (GE Healthcare). The gene encoding (*S*)-3-hydroxybutyryl-CoA dehydrogenase (Nmar_1028) was amplified by Q5 High-Fidelity DNA Polymerase (NEB, Frankfurt, Germany) from genomic DNA of *N. maritimus* using the forward primer (5′-GCG GCC ATA TCG AAG GTC GT*C ATA TG*G CAG TAA AAA ATA TCA CAA TTT TAG-3′) introducing an *Nde*I restriction site (italicized) at the initiation codon and the reverse primer (5′- TCT CAT GTT TGA CAG CTT ATC ATC GAT *AAG CTT* GAT TCA CTG CAT TGA TGT GAG-3′) introducing a *Hin*dIII site (italicized) after the stop codon. Touchdown PCR was used as follows: initial denaturation 3 min at 98°C, 20 cycles of 30-s denaturation at 98°C, 30-s primer annealing at 57°C (gradually reduced 0.2°C per cycle) and 40-s elongation at 72°C, followed by 15 cycles of 30-s denaturation at 98°C, 30-s primer annealing at 53°C and 40-s elongation at 72°C. The PCR product was treated with the corresponding restriction enzymes and then the gene was ligated into pET16b using T4 DNA ligase (NEB). The cloning of a gene into this vector with *Nde*I and *Hin*dIII restrictases adds an N-terminal His_10_-tag to the produced protein. The obtained plasmid was transformed into *E. coli* TOP10 for amplification.

### Heterologous Expression in *E. coli*

The recombinant vector pET16b-Nmar_1028 was transformed into *E. coli* Rosetta 2 (DE3). The cells were grown at 37°C in LB medium with 100 μg ampicillin ml^–1^ and 34 μg chloramphenicol ml^–1^. Expression of Nmar_1028 was induced at optical density (OD_578nm_) of 0.6 with 0.2 mM isopropyl-β-D-thiogalactopyranoside (IPTG), and the temperature was lowered to 16°C. The cells were harvested after additional growth for 15 h and stored at -20°C until use.

### Preparation of Cell Extracts

Frozen cells were suspended in a double volume of 20 mM Tris-HCl (pH 7.8) containing 0.1 mg ml^–1^ DNase I. The cell suspension was sonicated for 60 cycles of 1-s pulse and 2-s pause, and the cell lysate was centrifuged for 20 min (14,000 rpm; 4°C). The supernatant (cell extract) was used for protein purification immediately.

### Purification of Recombinant Protein Nmar_1028

The heterologously produced histidine-tagged (his-tagged) Nmar_1028 was purified using affinity chromatography. The supernatant was applied to 0.2-ml HisPur Ni-NTA Spin Column (Thermo Fisher Scientific, Rockford, United States) that had been equilibrated with 20 mM Tris-HCl containing 100 mM KCl (pH 7.8). The unwanted proteins were washed out with the same buffer containing 100 mM imidazole and the enzyme was eluted with the same buffer containing 500 mM imidazole. The recombinant protein was stored in 50% glycerol at -20°C after concentration using 10K Vivaspin Turbo 4 (Sartorius, Göttingen, Germany).

### Size Exclusion Chromatography

The native molecular mass of Nmar_1028 was determined by gel filtration on a Superdex 200 Increase column (24-ml volume; Cytiva) at a flow rate of 0.75 ml min^–1^. The column was equilibrated with buffer containing 20 mM Tris-HCl (pH 7.8) and 150 mM KCl. To make a standard curve, apoferritin (horse spleen, 443 kDa), alcohol dehydrogenase (yeast, 150 kDa), bovine serum albumin (BSA, 66 kDa), and carbonic anhydrase (bovine erythrocytes, 29 kDa) were used. The eluted fractions were stored in 50% glycerol at -20°C.

### Enzyme Assays

All enzyme activities of Nmar_1028 were measured spectrophotometrically in 300 μl reaction mixture at 30°C at 365 nm (ε_*NADH*_ = 3.4 mM^–1^ cm^–1^, ε_*NADPH*_ = 3.5 mM^–1^ cm^–1^; Bergmeyer, 1975). (*S*)-3-hydroxybutyryl-CoA dehydrogenase activity was detected in 100 mM Tris-HCl (pH 7.8) containing 0.5 mM NAD(P), 0.2 mM (*S*)-3-hydroxybutyryl-CoA, and purified enzyme. The concentration of (*S*)-3-hydroxybutyryl-CoA was varied (0.005–0.2 mM) for *K*_*m*_ determination. Activity of (*S*)-3-hydroxybutyryl-CoA dehydrogenase in the direction of NAD(P)H oxidation was measured in 100 mM Tris-HCl (pH 7.8) containing 0.5 mM NAD(P)H, 0.2 mM acetoacetyl-CoA and purified enzyme. The concentration of acetoacetyl-CoA was varied (0.005–0.2 mM) for *K*_*m*_ determination. To illustrate the effect of pH on enzyme activity, 50 mM MOPS/50 mM Tris (pH 7.0, 7.4) and 100 mM Tris (7.8, 8.0, 8.6, and 9.0) were used. One enzyme unit (U) corresponds to 1 μmol substrate converted per minute.

### Database Search and Phylogenetic Analysis

Query sequences for the database searches were obtained from NCBI data base. The BLASTP searches were performed via NCBI BLAST server^1^ (Altschul et al., 1990). The phylogenetic tree was constructed by using the maximum likelihood method and Jones-Taylor-Thornton (JTT) matrix-based model (Jones et al., 1992) in MEGA-X (Kumar et al., 2018). 145 amino acid sequences were involved in this analysis. All positions containing gaps were completely deleted. The GenBank accession numbers for the protein sequences are listed in Supplementary Table 1.

### Other Methods

DNA sequence determination of purified plasmids was performed by Eurofins (Ebersberg, Germany). Protein concentration was measured according to the Bradford method (Bradford, 1976) using BSA as a standard. Sodium dodecyl sulfate-polyacrylamide gel electrophoresis (SDS-PAGE; 12.5%) was performed as previously described (Laemmli, 1970). Proteins were visualized using Coomassie blue staining (Zehr et al., 1989). Protein identification was performed at the IZKF Core Unit Proteomics Münster based on tryptic in-gel digestion and mass spectrometric analysis using Synapt G2 Si coupled to M-Class (Waters Corp.). *K*_*m*_ and *V*_*max*_ values were calculated by GraphPad Prism5 software.

## Results

### Characterization of Recombinant Nmar_1028

**FIGURE 2:**
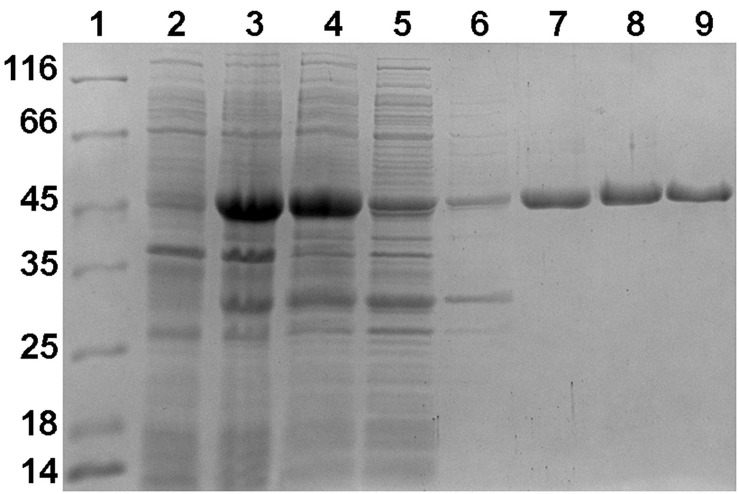
SDS-PAGE (12.5%) of fractions obtained during purification of heterologously produced Nmar_1028. Lane 1, molecular-weight markers (labeled in kDa); lane 2, cells before induction; lane 3, cells after induction; lane 4, cell extract; lane 5, flowthrough from Ni-NTA column; lane 6, wash with 50 mM imidazole; lane 7, elution with 500 mM imidazole; lane 8, the first peak during gel filtration; lane 9, the second peak during gel filtration.

Using a bioinformatic analysis, Nmar_1028 was identified as a putative (*S*)-3-hydroxybutyryl-CoA dehydrogenase in *N. maritimus* (Könneke et al., 2014). Here, we amplified the gene *nmar_1028*, cloned it in pET16b vector (resulting in the vector pET16b-Nmar_1028), and heterologously expressed it in *E. coli*. The recombinant protein was present in the soluble protein fraction (Figure 2). The molecular weight of purified protein on SDS gel (45 kDa) was close to the predicted size (44.8 kDa). The identity of the obtained protein was confirmed using tryptic in-gel digestion and mass spectrometric analysis (PLGS score: 12489.25; peptides: 90; theoretical peptides: 40; coverage: 98%; precursor RMS mass error: 10.1806 ppm; products: 1168; products RMS mass error: 10.8739 ppm; products RMS RT error: 0.01313549 min). Gel filtration chromatography showed the enzyme was eluted in two peaks. The molecular weight of these two peaks were estimated as 89.9 and 43.8 kDa, respectively, indicating that the enzyme was present in dimeric and monomeric forms in the preparation.

The specific activity of the protein after His-tag purification in (*S*)-3-hydroxybutyryl-CoA dehydrogenase reaction was 36.2 ± 1.9 U mg^–1^ protein, and the corresponding activities for the dimeric and monomeric forms of enzyme were 4.6 ± 0.4 U mg^–1^ protein and 69.6 ± 1.0 U mg^–1^ protein, respectively. Therefore, the activity of the dimeric protein was only 6.6% compared to the activity of the monomeric one. Only the monomeric enzyme was used for the following measurements of the dehydrogenase activity (Table 1). The enzyme was specific for NAD with high *V*_*max*_ and low *K*_*m*_ values for (*S*)-3-hydroxybutyryl-CoA (98.6 U mg^–1^ protein and 19 μM, respectively, as measured with 2 mM NAD). The activity with 0.5 mM NAD was slightly lower (Table 1). Only low activity with 3-hydroxypropionyl-CoA was detected (0.02 U mg^–1^ protein), confirming high specificity of the enzyme toward (*S*)-3-hydroxybutyryl-CoA. Besides NAD, the enzyme is able to use NADP as an electron acceptor. Nevertheless, its catalytic efficiency (*k*_*cat*_/*K*_*m*_) for NAD was 200 times higher than that for NADP (Table 1), being consistent with the published data for 3-hydroxybutyryl-CoA dehydrogenases. Apart from the *Clostridium kluyveri* enzyme (Madan et al., 1973), all other 3-hydroxybutyryl-CoA dehydrogenases characterized so far were (best) active with NAD. Although the capability to use NADP as an alternative coenzyme was observed in some cases, the catalytic efficiency for this cofactor was very low (Osumi and Hashimoto, 1980; Liu et al., 2020).

**TABLE 1:** Catalytic properties of heterologously produced (*S*)-3-hydroxybutyryl-CoA dehydrogenase Nmar_1028.

Substrate	*V*_*max*_ (U mg^–1^ protein)	*K*_*m*_ (mM)	*k*_*cat*_/*K*_*m*_ (s^–1^ mM^–1^)
**Forward reaction *^*a*^***			
(*S*)-3-Hydroxybutyryl-CoA (with 2 mM NAD)	98.6 ± 3.3	0.019 ± 0.002	3877
(*S*)-3-Hydroxybutyryl-CoA (with 0.5 mM NAD)	76.1 ± 3.4	0.017 ± 0.003	3344
NAD (with 0.2 mM (*S*)-3-hydroxybutyryl-CoA)	84.4 ± 2.8	0.10 ± 0.02	630
NADP (with 0.2 mM (*S*)-3-hydroxybutyryl-CoA)	10.1 ± 0.9	2.92 ± 0.63	3
**Reverse reaction*^*b*^***			
Acetoacetyl-CoA (with 0.5 mM NADH)	144.8 ± 7.7	0.026 ± 0.004	4045
NADH (with 0.2 mM acetoacetyl-CoA)	112.9 ± 7.3	0.016 ± 0.006	5271

**^*a*^No activity was detected with (R)-3-hydroxybutyryl-CoA (detection limit ≤ 0.012 U mg*^–^*^1^). The specific activity was 0.02 U mg*^–^*^1^ protein with 3-hydroxypropionyl-CoA. ^*b*^≤ 0.006 U mg*^–^*^1^ protein with 5 mM NADPH.**

The activity of Nmar_1028 was also measured in the reverse direction with NADH as acetoacetyl-CoA reduction to (*S*)-3-hydroxybutyryl-CoA (Table 1). The corresponding *V*_*max*_ and *K*_*m*_ values of the enzyme were 144.8 U mg^–1^ protein and 26 μM, respectively (with 0.5 mM NADH). The *k*_*cat*_/*K*_*m*_ value for NADH was 5,271 s^–1^ mM^–1^, no activity was detected with 5 mM NADPH, revealing NADH as the electron donor for the enzyme.

**FIGURE 3:**
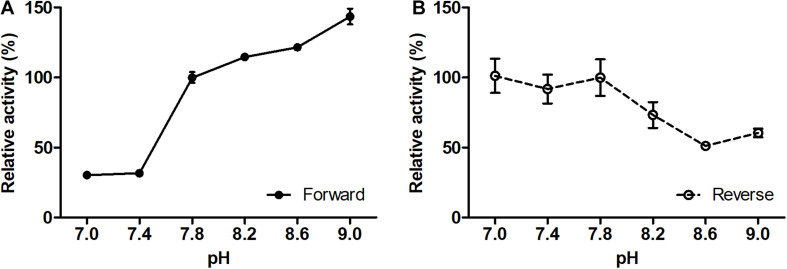
Effect of pH on the forward **(A)** and reverse **(B)** reaction rates of recombinant (*S*)-3-hydroxybutyryl-CoA dehydrogenase Nmar_1028. **(A)** 100% activity corresponds to 69.6 ± 2.7 U mg^– 1^ protein measured with 0.2 mM (*S*)-3-hydroxybutyryl-CoA and 0.5 mM NAD^+^ at pH 7.8. **(B)** 100% activity corresponds to 54.9 ± 7.2 U mg^– 1^ protein measured with 0.2 mM acetoacetyl-CoA and 0.5 mM NADH at pH 7.8. Values are means ± standard deviations of results from three independent measurements.

The (*S*)-3-hydroxybutyryl-CoA dehydrogenase activity of Nmar_1028 was affected by changes in pH (Figure 3), gradually increasing with increase of pH from 7 to 9 with ∼30% activity at pH 7.0 or 7.4 in comparison to 100% activity at pH 9. On the opposite, the activity for the reverse reaction was maximal in the buffers with lower pH (7.0–7.8), with remarkable activity decrease with the pH change from 7.8 to 9.0 (Figure 3). These results are typical for NAD-dependent dehydrogenases, as NAD^+^ reduction leads to the formation of NADH and H^+^.

As the *Mycobacterium tuberculosis* 3-hydroxybutyryl-CoA dehydrogenase activity was enhanced by divalent metals (Taylor et al., 2010), we performed the corresponding assays with the *N. maritimus* protein. Nmar_1028 did not require addition of divalent metals in the reaction mixture for activity (Table 2). The incubation of the enzyme in the presence of divalent metals resulted rather in inhibition of its activity. Whereas Mg^2+^, Mn^2+^, Ni^2+^, and Co^2+^ had only subtle effect on the activity, the addition of Fe^2+^ and Zn^2+^ strongly affected the Nmar_1028 activity.

**TABLE 2:** Influence of divalent cations on the activity of recombinant (*S*)-3-hydroxybutyryl-CoA dehydrogenase Nmar_1028.

Substrate	Relative activity (%)
No addition	100^*a*^
EDTA (0.5 mM)	100 ± 1
MgCl_2_ (5 mM)	90 ± 10
MnCl_2_ (5 mM)	88 ± 2
NiCl_2_ (1 mM)	79 ± 8
CoCl_2_ (1 mM)	76 ± 5
FeCl_2_ (1 mM)	35 ± 4
ZnCl_2_ (1 mM)	1.5 ± 0.3

**^*a*^100% activity corresponds to 69.6 ± 1.4 U mg*^–^*^1^ protein measured with 0.2 mM (S)-3-hydroxybutyryl-CoA and 0.5 mM NAD.**

### Distribution and Phylogenetic Analysis of *N. maritimus* (*S*)-3-Hydroxybutyryl-CoA Dehydrogenase

Whereas Nmar_1028 is the only homolog for (*S*)-3-hydroxybutyryl-CoA dehydrogenase in the genome of *N. maritimus*, several phylogenetically related proteins can catalyze this reaction in *M. sedula* (Liu et al., 2020). Msed_0389 has only low (*S*)-3-hydroxybutyryl-CoA dehydrogenase activity. The activity of bifunctional crotonyl-CoA hydratase/(*S*)-3-hydroxybutyryl-CoA dehydrogenase Msed_0399 is comparable to that of Nmar_1028, while its *K*_*m*_ value is 6 times higher than that of Nmar_1028 (Tables 1, 3). Msed_1423 is the main (*S*)-3-hydroxybutyryl-CoA dehydrogenase in *M. sedula* with the highest *V*_*max*_ value (96 U mg^–1^ protein) and the lowest *K*_*m*_ value (5 μM) to its substrate (Table 3). The catalytic properties of Nmar_1028 are rather comparable with those of Msed_1423 than of Msed_0399. Indeed, the *k*_*cat*_/*K*_*m*_ values of Nmar_1028 and Msed_1423 were 5- and 20-fold higher than the corresponding value of Msed_0399 in the (*S*)-3-hydroxybutyryl-CoA dehydrogenase reaction (Tables 1, 3).

**TABLE 3:** Catalytic properties of various so-far-characterized 3-hydroxyacyl-CoA dehydrogenases toward 3-hydroxybutyryl-CoA and/or acetoacetyl-CoA.

Organism	Protein	Gene	3-Hydroxybutyryl-CoA			Acetoacetyl-CoA			References
			*V_*max*_/k_*cat*_*	*K*_*m*_	*k*_*cat*_/*K*_*m*_	*V_*max*_/k_*cat*_*	*K*_*m*_	*k*_*cat*_/*K*_*m*_	
*Nitrosopumilus maritimus* SCM1	HBDH	Nmar_1028	98.6/74 (2 mM NAD) 76.1/57 (0.5 mM NAD)	0.019 (2 mM NAD)0.017 (0.5 mM NAD)	3,877 (2 mM NAD) 3,344 (0.5 mM NAD)	144.8/108	0.026	4,160	This work
*Metallosphaera sedula* DSM 5348	HBDH	Msed_1423	96/75(0.5 mM NAD)	0.005	14,957	NR	NR	NR	Liu et al., 2020
	CCH/HBDH	Msed_0399	76/90^*a*^ 22.6/19^*a*^ 27.6/33^*a*^	0.12 0.060.2	748 445163	NR	NR	NR	Ramos-Vera et al., 2011; Hawkins et al., 2014; Liu et al., 2020
*Ignicoccus hospitalis*	CCH/HBDH	Igni_1058	117/152	0.086	1,800	NR	NR	NR	Flechsler et al., 2021
*Cupriavidus necator* H16	HBDH/enoyl-CoA hydratase^*b*^	H16_A0461	NR	NR	NR	149/216	0.048	4,500	Volodina and Steinbüchel, 2014
	HBDH	RePaaH1	NR	NR	NR	85.4/46	0.018	2,530	Kim J. et al., 2014
*Clostridium butyricum*	HBDH	CbHBD	NR	NR	NR	56.8/29	0.028	1,035	Kim E. J. et al., 2014
*Caenorhabditis elegans* Bristol N2	HADH	cHAD	NR	NR	NR	78/45	0.072	600	Xu et al., 2014
*Mycobacterium tuberculosis* H37Rv	HBDH	FadB2	0.19/0.096	0.0435	2	0.13/0.065	0.0656	1	Taylor et al., 2010
*Escherichia coli* ATCC 11775	Fatty acid oxidation complex	?	NR	NR	NR	64/288	0.066	4,364	Binstock and Schulz, 1981
*Mycolicibacterium smegmatis* ATCC 14468	HADH	?	NR	NR	NR	12.5/10	0.036	291	Shimakata et al., 1979
*Homo sapiens*	Short-chain HADH I	SCHAD I	NR/119	0.0225	5,290	NR/1,287	0.0166	77,500	Filling et al., 2008
	Short-chain HADH II	SCHAD II	NR/4.83	0.0852	58	NR/23.8	0.0257	927	Filling et al., 2008
	Short chain L-HADH	rSCHADH6	NR	NR	NR	459/252	0.0187	13,500	Barycki et al., 1999
	Short chain L-HADH	SCHAD	NR/0.011	0.043	0.26	NR	NR	NR	He et al., 1999
	L-HADH	HBHAD	NR	NR	NR	NR/37	0.089	416	He et al., 1998
*Rattus norvegicus*	HADH	HAD	NR	NR	NR	976/569	0.044	13,000	Liu et al., 2004
	HADH I	?	270/144	0.0699	2,060	820/437	0.0169	25,878	Osumi and Hashimoto, 1980
	HADH II	?	15.6/20	0.0422	474	23.5/30	0.0397	760	Osumi and Hashimoto, 1980
*Sus scrofa*	L-HADH	HAD	NR	NR	NR	NR/330	0.003	110,000	He and Yang, 1998
	L-HADH	?	39/21	0.0072	2,889	230/123	0.0118	10,395	He et al., 1989
	L-HADH	?	NR	NR	NR	426/227	0.06	3,787	Noyes and Bradshaw, 1973

**V_*max*_, U mg*^–^*^1^ protein; k_*cat*_, s*^–^*^1^; K_*m*_, mM. (S)-3-hydroxybutyryl-CoA dehydrogenase, HBDH; crotonyl-CoA hydratase, CCH; 3-hydroxyacyl-CoA dehydrogenase, HADH?, unknown; NR, not reported. ^*a*^The V_*max*_ values are normalized to 75°C based on the assumption that a 10°C rise in temperature doubles the reaction rate. ^*b*^Specific activity with crotonyl-CoA was 98 U mg*^–^*^1^.**

**FIGURE 4:**
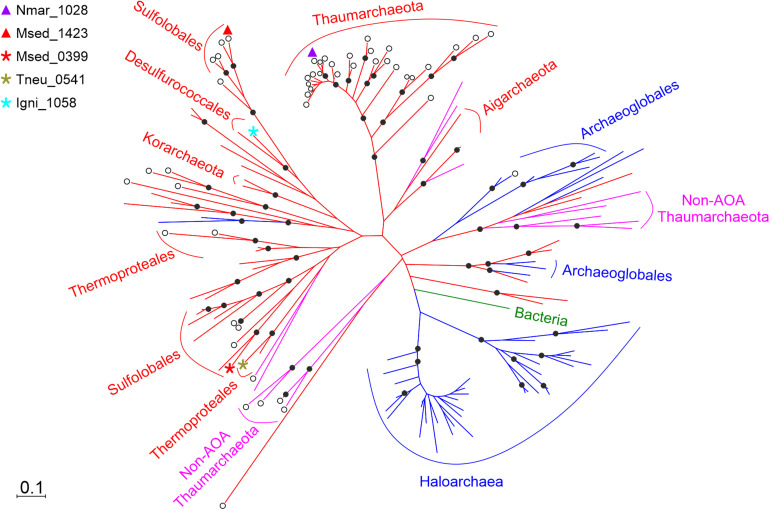
Phylogenetic tree of (*S*)-3-hydroxybutyryl-CoA dehydrogenase Nmar_1028. The sequences are marked as following: purple triangle, Nmar_1028; red triangle, Msed_1423; red star, Msed_0399; olive star, Tneu_0541 (from *Pyrobaculum neutrophilum*); turquoise star, Igni_1058 (from *Ignicoccus hospitalis*); ◯, dehydrogenase; without ◯, fusion protein; 80 sequences from TACK group in red, 15 sequences from non-AOA *Thaumarchaeota* in fuchsia, 49 sequences from *Euryarchaeota* in blue, 1 sequence from bacteria in green. The scale bar represents a difference of 0.1 substitutions per site. The percentage bootstrap values for the clade of this group were calculated in 1000 replications. Only values above 70% are shown (as ⬤). The accession numbers are listed in Supplementary Table 1. The phylogenetic tree generated by maximum-parsimony algorithm (data not shown) was similar with minor exceptions.

To trace the evolution of the HP/HB cycle, we performed a comparative phylogenetic analysis (Figure 4) of the corresponding enzymes. The homologs of (*S*)-3-hydroxybutyryl-CoA dehydrogenase Nmar_1028 were found in the genomes of all sequenced AOA, suggesting that all these archaea share the ability to grow autotrophically by using the HP/HB cycle. Moreover, these homologs formed a separate cluster containing the sequences of AOA, which is phylogenetically separated from the cluster of the corresponding crenarchaeal sequences (Figure 4). Interestingly, Nmar_1028 and Msed_1423 share 42/60% of sequence identity/similarity, whereas Nmar_1028 and the dehydrogenase domain of Msed_0399 share 41/62% of sequence identity/similarity (according to the BLASTP search). It appears that despite their homology, these three proteins evolved independently from each other, confirming the proposed convergent evolution of HP/HB cycle in Cren- and Thaumarchaeota.

## Discussion

Aerobic ammonia oxidation is a crucial part of the modern nitrogen cycle and an environmentally important process that is responsible for a large part of dark ocean inorganic carbon fixation (Meador et al., 2020). This process is usually dated back to the great oxidation event (Ren et al., 2019), and its obligate requirement for oxygen can be accounted for by the fact that the first reaction in ammonia oxidation is performed via ammonia monooxygenase. It can be found only in a limited number of bacteria and archaea. A recent phylogenomic analysis suggested that AOA originated from the terrestrial non-ammonia-oxidizing ancestors and expanded to the shallow and then deep ocean upon their oxygenation (Ren et al., 2019), while ammonia-oxidizing bacteria are much younger (Ward et al., 2021). Note however that this model is still under discussion (Abby et al., 2020). The absence of the key genes of the HP/HB cycle in the non-ammonia-oxidizing Thaumarchaeota (non-AOA) and their presence in all AOA studied so far (Ren et al., 2019) suggests that the thaumarchaeal HP/HB cycle evolved in AOA ancestors *de novo*, and that the last common ancestor of AOA had already a fully evolved HP/HB cycle. Our analysis also confirms this model, showing the (*S*)-3-hydroxybutyryl-CoA dehydrogenase studied here is present in all studied AOA, but not in non-AOA (Figure 4). Similarly, a 3-hydroxypropionyl-CoA dehydratase/crotonyl-CoA hydratase gene was found only in ammonia-oxidizing representatives of the phylum Thaumarchaeota (Liu et al., 2021).

The HP/HB cycle exists in two variants, and the apparent similarity in the intermediates of the cycle in AOA and in Sulfolobales is in sharp contrast with the results of the biochemical and/or phylogenetic analysis of the corresponding enzymes (Könneke et al., 2014; Otte et al., 2015; Liu et al., 2021). The difference in the autotrophic pathways in these two archaeal groups goes along with the difference in their ecology and environmental requirements. While Sulfolobales like *M. sedula* gain energy from the highly exergonic aerobic hydrogen oxidation, AOA use a much less energetically favorable reaction, the oxidation of ammonia to nitrite. Moreover, some AOA are adapted to perform near maximum autotrophic growth at nanomolar concentrations of ammonia (Martens-Habbena et al., 2009), further highlighting the high efficiency of their metabolism. Correspondingly, AOA and Sulfolobales convergently evolved the pathways that are best adapted to their needs, with a less energetically efficient but kinetically superior variant functioning in Sulfolobales (Könneke et al., 2014).

(*S*)-3-Hydroxybutyryl-CoA dehydrogenases in *M. sedula* and *N. maritimus* are both homologous to the dehydrogenase domain of crotonyl-CoA hydratase/(*S*)-3-hydroxybutyryl-CoA dehydrogenase (though to different clusters in the tree, Figure 4). This fusion protein is present in many bacteria and archaea and was predicted to be present in the common ancestor of Cren- and Thaumarchaeota (Williams et al., 2017). Apparently, the protein lost its dehydratase domain in both archaeal groups in the course of evolution, probably because of its redundancy. Indeed, the HP/HB cycle possesses two very similar (de)hydratase reactions, crotonyl-CoA hydratase and 3-hydroxypropionyl-CoA dehydratase. To catalyze these reactions, both AOA and Sulfolobales possess a promiscuous 3-hydroxypropionyl-CoA dehydratase/crotonyl-CoA hydratase that was laterally transferred from bacteria to their ancestors (Liu et al., 2021). The importance of the acquisition of bacterial genes for the evolution of AOA has been shown earlier (Deschamps et al., 2014).

The apparent independent loss of the enoyl-CoA hydratase domain in the ancestors of AOA and Sulfolobales suggests that the separation of the hydratase and dehydrogenase reactions should give certain advantages for organisms using the HP/HB cycle. What could it be? The crotonyl-CoA hydratase/(*S*)-3-hydroxybutyryl-CoA dehydrogenase Msed_0399 is only weakly active with 3-hydroxypropionyl-CoA. We suggest that an acquisition of a promiscuous bacterial hydratase capable of catalyzing both of these reactions with high efficiency made the dehydratase domain of a fusion protein redundant, leading to its loss. In addition, the analysis of catalytic properties of different (*S*)-3-hydroxybutyryl-CoA dehydrogenases suggests that the fusion proteins have much higher *K*_*m*_ values to (*S*)-3-hydroxybutyryl-CoA than the stand-alone dehydrogenases (Table 3). In fact, a two−step reaction catalyzed by a fusion enzyme usually demonstrates an improved catalytic efficiency, which is usually attributed to a substrate channeling, however only if the intermediate concentration remains low (Rabe et al., 2017). As the *K*_*m*_ value to (*S*)-3-hydroxybutyryl-CoA in the dehydrogenase reaction is relatively high, this factor is probably not relevant for the functioning of the HP/HB cycle, and the merging of the dehydrogenase and hydratase in a fusion protein for the HP/HB cycle becomes dispensable.

Interestingly, autotrophic Crenarchaeota of the orders Thermoproteales and Desulfurococcales use the fusion protein to catalyze the crotonyl-CoA hydratase and (*S*)-3-hydroxybutyryl-CoA dehydrogenase reactions in the DC/HB cycle (Huber et al., 2008; Ramos-Vera et al., 2009; Flechsler et al., 2021). This cycle does not require a 3-hydroxypropionyl-CoA dehydratase reaction. Furthermore, the crotonyl-CoA hydratase activity of the fusion protein is high (Ramos-Vera et al., 2009; Hawkins et al., 2014; Flechsler et al., 2021), making the tinkering around this reaction unnecessary.

The reaction studied here is neither unique nor characteristic for the HP/HB cycle. Nevertheless, the enzymatic conversion of (*S*)-3-hydroxybutyryl-CoA to acetoacetyl-CoA is essential for the functioning of the cycle. The genome of *N. maritimus* is streamlined, and Nmar_1028 appears to be the only (*S*)-3-hydroxybutyryl-CoA dehydrogenase in this important marine archaeon. Moreover, some autotrophic Sulfolobales also have only one enzyme to catalyze this conversion (Liu et al., 2020), what makes it as essential for the functioning of the cycle as any other enzyme involved in the cycle. Apart from autotrophic CO_2_ fixation in the HP/HB and DC/HB cycles, the enzyme participates in various metabolic process like β-oxidation, polyhydroxyalkanoate metabolism, or fermentations. The low *K*_*m*_ values of *N. maritimus* and *M. sedula* enzymes to (*S*)-3-hydroxybutyryl-CoA suggest their adaptation to the functioning in the HP/HB cycle. Moreover, these enzymes are of biotechnological interest and could be applied for the biosynthesis of e.g., *n*-butanol (Kim and Kim, 2014). The HP/HB cycle is a promising candidate for microbial production of chemicals from CO_2_ (Loder et al., 2016), and the studied enzyme can be used e.g., to the reconstitution of the HP/HB cycle or similar process in a proper mesophilic host organism (Fuchs and Berg, 2014).

## Data Availability Statement

The original contributions presented in the study are included in the article/Supplementary Material, further inquiries can be directed to the corresponding author/s.

## Author Contributions

IB designed the experiments. LL, DS, and MK performed the experiments. LL, DS, and IB analyzed the data. LL and IB wrote the manuscript. All authors read and approved the final manuscript.

## Conflict of Interest

The authors declare that the research was conducted in the absence of any commercial or financial relationships that could be construed as a potential conflict of interest.

## Footnotes

1 http://www.ncbi.nlm.nih.gov/BLAST/
