# Supplementary material for: (S)-3-Hydroxybutyryl-CoA Dehydrogenase From the Autotrophic 3-Hydroxypropionate/4-Hydroxybutyrate Cycle in Nitrosopumilus maritimus
Source: Front Microbiol. 2021 Jul 5;12:712030. doi: 10.3389/fmicb.2021.712030 (PMC8287830; doi:10.3389/fmicb.2021.712030)
Supplement: Supplementary file 1 [file Table_1.docx]

**Supplementary Tables.**

**Table S1**. The GenBank accession numbers for the sequences used for the construction of the phylogenetic tree shown in the **Figure 4**.

| Genome Name | GenBank No. |
| --- | --- |
| *Aigarchaeota* archaeon NZ13_MG1 | PUA34257.1 |
| *Candidatus* Caldiarchaeum subterraneum | BAJ47153.1 |
| *Candidatus* Caldiarchaeum subterraneum | BAJ50004.1 |
| *Candidatus* Caldiarchaeum subterraneum | BAJ49086.1 |
| *Candidatus* Caldiarchaeum subterraneum | BAJ46931.1 |
| *Candidatus* Bathyarchaeota archaeon | RLI15722.1 |
| *Candidatus* Bathyarchaeota archaeon | RLI26916.1 |
| *Candidatus* Bathyarchaeota archaeon | RLI47538.1 |
| *Candidatus* Bathyarchaeota archaeon | TFH19338.1 |
| *Candidatus* Bathyarchaeota archaeon RBG_13_52_12 | OGD56491.1 |
| *Acidilobus saccharovorans* 345-15 | ADL19436.1 |
| *Aeropyrum camini* JCM 12091 | BAN90403.1 |
| *Caldisphaera lagunensis* DSM 15908 | AFZ69922.1 |
| *Caldisphaera lagunensis* DSM 15908 | AFZ71226.1 |
| *Caldisphaera lagunensis* DSM 15908 | AFZ70269.1 |
| *Caldivirga maquilingensis* IC-167 | ABW01183.1 |
| *Ignicoccus hospitalis* DSM 18386 | ABU82236.1 |
| *Metallosphaera cuprina* Ar-4 | AEB95783.1 |
| *Metallosphaera sedula* DSM 5348 | ABP94576.1 |
| *Pyrobaculum oguniense* DSM 13380 | AFA39936.1 |
| *Pyrodictium delaneyi* Su06 | ALL00473.1 |
| *Pyrolobus fumarii* DSM 11204 | AEM39286.1 |
| *Saccharolobus solfataricus* P2 | AAK42645.1 |
| *Sulfolobus acidocaldarius* DSM 639 | AAY80486.1 |
| *Sulfolobus acidocaldarius* DSM 639 | AAY81504.1 |
| *Sulfolobus acidocaldarius* DSM 639 | AAY80461.1 |
| *Sulfolobus acidocaldarius* DSM 639 | AAY81494.1 |
| *Sulfolobus islandicus* L.S.2.15 | ACP34483.1 |
| *Sulfolobus islandicus* L.S.2.15 | ACP34844.1 |
| *Sulfurisphaera tokodaii* 7 | BAK54139.1 |
| *Pyrobaculum neutrophilum* V24Sta | ACB39484.1 |
| *Vulcanisaeta distributa* DSM 14429 | ADN51296.1 |
| *Vulcanisaeta distributa* DSM 14429 | ADN49667.1 |
| *Vulcanisaeta moutnovskia* 768-28 | ADY00549.1 |
| *Vulcanisaeta moutnovskia* 768-28 | ADY01580.1 |
| *Candidatus* Korarchaeota archaeon NZ13-K | RDD53934.1 |
| *Candidatus* Korarchaeum cryptofilum OPF8 | ACB06874.1 |
| *Candidatus* Methanodesulfokores washburnensis | WP_125671879.1 |
| *Archaeoglobus sulfaticallidus* | WP_015591018.1 |
| *Geoglobus acetivorans* | WP_048091314.1 |
| *Archaeoglobus fulgidus* DSM 4304 | AAB90118.1 |
| *Archaeoglobus fulgidus* DSM 4304 | AAB91209.1 |
| *Archaeoglobus fulgidus* DSM 4304 | AAB88983.1 |
| *Archaeoglobus fulgidus* DSM 4304 | AAB90948.1 |
| *Ferroglobus placidus* DSM 10642 | ADC65197.1 |
| *Ferroglobus placidus* DSM 10642 | ADC66080.1 |
| *Ferroplasma acidarmanus* Fer1 | ZP_00608931.1 |
| *Geoglobus ahangari* 234 | WP_052747724.1 |
| *Geoglobus ahangari* 234 | WP_048095968.1 |
| *Halalkalicoccus jeotgali* DSM 18796 | ADJ15163.1 |
| *Halalkalicoccus jeotgali* DSM 18796 | ADJ16611.1 |
| *Haloarcula hispanica* CGMCC 1.2049 | AEM57293.1 |
| *Haloarcula hispanica* CGMCC 1.2049 | AEM58074.1 |
| *Haloarcula marismortui* ATCC 43049 | AAV46045.1 |
| *Haloarcula marismortui* ATCC 43049 | AAV46847.1 |
| *Halobacterium salinarum* NRC-1 | AAG19656.1 |
| *Halobiforma lacisalsi* JCM 12983 | APW99729.1 |
| *Haloferax mediterranei* R-4 | AFK20711.1 |
| *Haloferax mediterranei* R-4 | AFK20502.1 |
| *Haloferax volcanii* ATCC 29605 | ADE04943.1 |
| *Haloferax volcanii* ATCC 29605 | ADE01983.1 |
| *Halogeometricum borinquense* DSM 11551 | ADQ69067.1 |
| *Halogeometricum borinquense* DSM 11551 | ADQ66878.1 |
| *Halohasta litchfieldiae* tADL | WP_089673896.1 |
| *Halomicrobium mukohataei* DSM 12286 | ACV48180.1 |
| *halophilic archaeon* DL31 | WP_014051246.1 |
| *Halopiger xanaduensis* SH-6 | AEH37211.1 |
| *Halorubrum lacusprofundi* ATCC 49239 | ACM57325.1 |
| *Halostagnicola larsenii* DSM 17691 | WP_049954865.1 |
| *Halostagnicola larsenii* DSM 17691 | AHF99941.1 |
| *Halostagnicola larsenii* DSM 17691 | AHF98285.1 |
| *Halostagnicola larsenii* DSM 17691 | AHF99098.1 |
| *Halovivax ruber* DSM 18193 | AGB15944.1 |
| *Natrialba magadii* ATCC 43099 | ADD06659.1 |
| *Natrialba magadii* ATCC 43099 | ADD04618.2 |
| *Natrinema pellirubrum* 157 | AGB30834.1 |
| *Natrinema pellirubrum* 157 | AGB32431.1 |
| *Natronobacterium gregoryi* SP2 | AFZ71725.1 |
| *Natronococcus occultus* DSM 3396 | AGB38518.1 |
| *Natronococcus occultus* DSM 3396 | AGB37124.1 |
| *Natronomonas pharaonis* DSM 2160 | CAI50252.1 |
| *Natronomonas pharaonis* DSM 2160 | CAI49468.1 |
| *Picrophilus torridus* DSM 9790 | AAT43606.1 |
| *Salinarchaeum* sp. Harcht-Bsk1 | WP_020445055.1 |
| *Salinarchaeum* sp. Harcht-Bsk1 | WP_020447818.1 |
| *Thermoplasma volcanium* GSS1 | BAB60475.1 |
| *Acidianus hospitalis* W1 | WP_013775903.1 |
| *Metallosphaera cuprina* Ar-4 | AEB95791.1 |
| *Metallosphaera cuprina* Ar-4 | AEB94917.1 |
| *Metallosphaera sedula* DSM 5348 | ABP94566.1 |
| *Metallosphaera sedula* DSM 5348 | ABP95581.1 |
| *Pyrobaculum oguniense* DSM 13380 | AFA39375.1 |
| *Pyrobaculum oguniense* DSM 13380 | AFA40703.1 |
| *Saccharolobus solfataricus* P2 | AAK42978.1 |
| *Saccharolobus solfataricus* P2 | AAK43102.1 |
| *Sulfolobus acidocaldarius* DSM 639 | AAY80435.1 |
| *Sulfolobus islandicus* L.S.2.15 | ACP36559.1 |
| *Sulfolobus islandicus* L.S.2.15 | ACP36484.1 |
| *Sulfurisphaera tokodaii* 7 | BAB67523.1 |
| *Sulfurisphaera tokodaii* 7 | BAK54732.1 |
| *Vulcanisaeta distributa* DSM 14429 | ADN50667.1 |
| *Candidatus* Nitrosopumilus koreensis AR1 | AFS81031.1 |
| *Candidatus* Nitrosopumilus sediminis AR2 | AFS83014.1 |
| *Candidatus* Nitrososphaera evergladensis SR1 | AIF84511.1 |
| *Candidatus* Nitrososphaera gargensis Ga9-2 | AFU58337.1 |
| *Cenarchaeum symbiosum* A | ABK77048.1 |
| *Nitrosopumilus maritimus* SCM1 | ABX12924.1 |
| *Nitrososphaera viennensis* EN76 | AIC15321.1 |
| *Archaeoglobus fulgidus* DSM 4304 | AAB90218.1 |
| *Candidatus* Acetothermum autotrophicum | BAL58613.1 |
| *Candidatus* Nitrosocaldus cavascurensis SCU2 | WP_103287567.1 |
| *Candidatus* Nitrosarchaeum limnium SFB1 | EGG42527.1 |
| *Candidatus* Nitrosomarinus catalina SPOT01 | WP_086907368.1 |
| *Candidatus* Nitrosopelagicus brevis CN25 | WP_048106914.1 |
| *Candidatus* Nitrosopumilus sp. SW | WP_141978191.1 |
| *Candidatus* Nitrosotenuis aquarius AQ6F | WP_100183388.1 |
| *Candidatus* Nitrosotenuis cloacae SAT1 | WP_048187691.1 |
| *Candidatus* Nitrosotenuis sp. DW1 | WP_179367921.1 |
| *Nitrosarchaeum* sp. AC2 | WP_179365039.1 |
| *Nitrosopumilus adriaticus* NF5 | WP_048118996.1 |
| *Nitrosopumilus cobalaminigenes* HCA1 | WP_179361499.1 |
| *Nitrosopumilus oxyclinae* HCE1 | WP_179363372.1 |
| *Nitrosopumilus piranensis* D3C | WP_148703206.1 |
| *Nitrosopumilus* sp. MH-Pat-all_metabat2_16 | QMU53494.1 |
| *Nitrosopumilus ureiphilus* PS0 | WP_179370863.1 |
| *Candidatus* Nitrosocosmicus exaquare G61 | WP_148686947.1 |
| *Candidatus* Nitrosocosmicus franklandus NFRAN1 | WP_134485287.1 |
| *Candidatus* Nitrosocosmicus oleophilus MY3 | ALI36443.1 |
| *Candidatus* Nitrosotalea devanaterra | CUR51822.1 |
| *Candidatus* Nitrosotalea okcheonensis NCS1 | WP_157926869.1 |
| *Thaumarchaeota* archaeon SAT139 | IMG_2787370918 |
| *Thaumarchaeota* archaeon SP3992 | IMG_2787363886 |
| *Thaumarchaeota* archaeon UBA223 | DAHJ01000013.1 MVLFVKRVGIVGGGAMGSQIADIMAINGKEVIIKDISEEFLKKARENVENNLDSLLEFNVTRADKEIERIEKTNQIKLTDEQKNKIKETLKPKFNEDMKKEAMDKIHTTTDYKEFNDVDLVIEAVLETVDLKRQVFTELDKNTPSHAILATNTSSLSVTEIASATSRPEKVVGVHFFNPPVTLPLVEVIPGMETSEETVNDMIDFMSTIRNHRYPMQPIKVKEVPGFLVNRILFAMMNEAYSCYDEGVASMRDIDLAMKAGAGMPMGPFELSDLVGIDVIYHVEEEVRKMIGGNTMRQPSQTIRKLYHAGRYGKKTKRGFYDYR |
| *Thaumarchaeota* YP1_bin3 | IMG_2718465564 |
| *Thaumarchaeota* Fn1 | IMG_2558669888 |
| *Thaumarchaeota* archaeon BS4 (Beowulf) | IMG_2519954921 |
| *Thaumarchaeota* archaeon DS1 (Dragon) | IMG_2263082618 |
| *Candidatus* Caldiarchaeum subterraneum | HHK68230.1 |
| *Thaumarchaeota* YP1_bin3 | IMG_2718464938 |
| *Thaumarchaeota* Fn1 | IMG_2558670860 |
| *Thaumarchaeota* archaeon bog_1367 | PLFZ01000160.1 MGHGISEAAALAGFDVTIYDVRQDFLDSGLEKIRWSVSKLEEKGVLSPGKGAEVLARIHTTLDLHAMAPLDLIIEAVPEELTVKTKVFQEIDRENKTAFLASNTSTIPITEIAAATSRPQRFVGIHFFNPPVLMPLVEVIRGELTDQETVDAAVSFSKALGKQVVLCRKDVPGFIVNRILGPLLNEAAWTVGRGQATVEQVDSAAVYKTGLPMGLFELADYTGIDVIYKAAEAVKSREPAALAPAPLFSEKYRQGKLGKKTGEGFYSYGKASGRPQITKEAGESVDPLSFFCVAVNAAAWLVRNQVCSEEDLDLAVKLGLGFPDGLLRMADRWGIDRIVAALREKQSLYGPEYAPDPLLEEMARSGSLGAATRKGFYDYSTSEKRLEELILRKAPPLAWVSLNRPHRLNTITPKMTEELEAAARDLAADGSIRVVILAGEGEKAFSAGADLTSFGFSSPVKAFDASRRMYEVFTLFETMPKPVIAAINGFAFGGGCELALACDFRLASESSQIGLTETSLGIIPGAGGTQRLLKLVGLAKAREMIYLGSRLSAGEALKAGLVDRVFANADFRSGVEEFANRLAKRAPLSLRFSKQALNVATQSPPNQGQYFEAGAFAMLLSTQDASEGITSFLSKKEPDFKGE |
| *Thaumarchaeota* archaeon palsa_1368 | PLCG01000092.1 LSSEARPAPTRVVSVAVIGAGTMGHGIAEVAALAGHPTVLYDVEARFLDSGMEKIKWSLSKLAEKGAITSEKAKQAAAGIVCTTDLSKAASCDLVIEAAPENLDIKRDLFARLDSLGKGSLLATNTSSIPISEIAAATSRPDMVVGIHFFNPPVMMPLVEVIRGAKTSDESVRRAAAFCEELGKRVVVCEKDVPGFIVNRVIGPLINEAAWVVARGEATITQVDSCCVHKVGLPMGLFELADFTGIDVIYAASQTMRSRDPAALPVATVLGEKTKAGKNGRKTGEGFYNYSSGGGASYSRTEGDSLDPLLIFSVGINSAAWLLRNQVCSKEDLDSSVKLGLGFPEGILQMADAWGIDKVVETLRKKQAAHGDSYAPDPLLVQMDERGDRGSRSGRGFYDYSSKETKLEEIVLKKAPPLAWITLSRVHRHNTITPKMMDELETVANDLARDGAVRVVIISGEGGKAFSAGADLTTFEFNSPLKAFEASRRMFEVFSLFEKMPKPVIAAINGYAFGGGCELALACDFRLASESSQIGLTETSLGIIPGAGGTQRLLRLVGLSKAKELIYFGERLPAQEALKAGLLDRVYANDSFQTEVEGFAKKLAKRAPVSLKLAKYAINLAQQVPNADAGQLFEAGG??IHFSPEHEDLRTWAREFANREVAPLAEKIDKSDEYPFGLAKKMGEYGLLGIGTPKEYGGLGLDLVSAVVVAEEIAKVSLSAGLIIGVQNGLVAYPISQFGSKEQKEKFLPRLVKGEIIGSYGLTEPGAG |
| *Thaumarchaeota* archaeon BS4 (Beowulf) | IMG_2519955681 |
| *Thaumarchaeota* archaeon DS1 (Dragon) | IMG_2263082095 |
| *Thaumarchaeota* archaeon UBA164 | DAJU01000010.1 MGHGIGEVFAIAGYRVSLIDVSDDALRKALDRIQESLNTLYQKGRIKEPVQDVMNRISTAKAIKDGVSDAGLVIEAVPEDYKIKVSVLSEAEKWAPEDAILATNTSNMLVSELAEPLKLKERFLGLHFFNPPVVMKLVEVIRGQHTSDNVYEVAYELVKGLGKVPVRVKDSVGFIVNRILAPELLYLCMLVDSGKVVPAEVDTFFRSQGMPMGLYELMDFVGLDVVYDSLKYYAEKLSPEYGKCKKIEEMVSSGLLGKKSGRGFYEWEGGKAKIPPAQPTEKVSLLDVMAIQVNEASKLLEAGIASPDDIETAVKLGLNLPFGPITVAKSVPAKDIKEKLEAISRELGCAIFEPTSSIKEGKLRELIEGRPVQEKKEVAQGPVVMKRVSERVVRVEINRPKLNLISPQVVEELERVVDQLASDRETWVVIISGAGENFSAGADLSTYFSDQVQFMEFSRKGERVFRKITELPQITIAEVKGYALGGGFELALACDIRVASQGSTLGFPEVTLGLVPGWGGTQRLPKLAGMSRALDLILTGRRISGAEAYQMGIVNRIIEGDPDQWTDRFAQELAGTVAPVAARLAKQLVNKASEVPMDIGLDMESTSFGVLFGTQDLKEGVSSLLQKRKPQYKGR |
